# Metastatic Patterns of Malignant Germ Cell Tumors Vary by Histologic Subtype and Primary Site

**DOI:** 10.3390/medicina61111990

**Published:** 2025-11-05

**Authors:** Hyung Kyu Park

**Affiliations:** Department of Pathology, Chungnam National University School of Medicine, Daejeon 35015, Republic of Korea; hkpark@g.cnu.ac.kr

**Keywords:** germ cell tumor, testicular germ cell tumor, germinoma, neoplasm metastasis, teratoma, embryonal carcinoma

## Abstract

*Background and Objectives*: Malignant germ cell tumors (GCTs) are rare but clinically significant neoplasms arising in gonadal and extragonadal sites. Malignant GCTs, divided into seminomatous and non-seminomatous subtypes, show diverse biological behavior. Although molecular studies have advanced understanding of their origins and genetic features, little is known about metastatic patterns due to their rarity and generally favorable outcomes. This study aimed to describe metastatic patterns of malignant GCTs across primary sites and histologic subtypes using population-based database. *Materials and Methods*: Data were extracted from the Surveillance, Epidemiology, and End Results (SEER) program for patients diagnosed with malignant GCTs between 2010 and 2022. Cases were stratified by primary site (testis, ovary, mediastinum), age group (<8 years vs. ≥8 years), and histologic subtype. Metastatic patterns were assessed using both overall and organotropic metastasis rates, and differences between groups were evaluated descriptively using appropriate statistical tests. *Results*: A total of 32,015 malignant GCTs were identified, comprising 93.0% testicular, 5.6% ovarian, and 1.4% mediastinal tumors. In patients aged ≥8 years, ovarian tumors tended to show generally lower lymph node and distant metastasis rates. In contrast, mediastinal tumors appeared to have the highest distant metastasis rates. Organotropic analysis suggested distinct subtype- and site-specific differences. For seminoma/dysgerminoma, the organotropic metastasis pattern was generally consistent across different primary sites, whereas the other subtypes showed variable organotropic metastasis rates depending on the primary site. *Conclusions*: The metastatic patterns of GCTs appear to differ by histologic subtype and primary site. These findings suggest that both subtype and site of origin should be considered when assessing metastatic risk and may provide a framework for improved risk stratification in clinical practice.

## 1. Introduction

Germ cell tumors (GCTs) are a unique and biologically heterogeneous group of neoplasms derived from the stem cells of the early embryo and the germ line [1]. These tumors most commonly arise in the gonads, with the testes in males and the ovaries in females being the predominant sites. However, they can also develop in extragonadal locations such as the mediastinum, retroperitoneum, sacrococcygeal region, and central nervous system, particularly along the midline of the body [2,3]. Although the majority of GCTs are benign, malignant forms are of considerable clinical significance. Testicular malignant GCTs are the most frequent solid tumors in males aged 15–40 years [4,5], whereas ovarian malignant GCTs, though less common overall, account for nearly 70% of ovarian cancers diagnosed in women younger than 30 years [6].

Histologically, malignant GCTs have traditionally been divided into seminomas and non-seminomas in the testis, and into dysgerminomas and non-dysgerminomas in the ovary [7,8,9]. Seminomas and dysgerminomas are morphologically identical tumors, differing only in nomenclature according to their gonadal site of origin. The non-seminomatous group encompasses yolk sac tumor (YST), embryonal carcinoma (EC), choriocarcinoma (CHC), and teratoma, while tumors composed of more than one element are referred to as mixed GCTs. Each subtype displays distinct clinical and biological behavior, and this classification has long provided a practical framework for diagnosis, treatment, and prognostication [10,11,12].

Recent molecular and genetic insights have further refined this framework, particularly in testicular tumors, which are the most common and extensively studied. Most testicular GCTs arise from a precursor lesion termed germ cell neoplasia in situ (GCNIS), which is typically associated with the gain of chromosome 12p, often in the form of isochromosome 12p [13,14]. In contrast, a subset of non-GCNIS–derived tumors, including certain teratomas, YSTs, and spermatocytic tumors, lacks this alteration [15]. Reflecting these findings, the current WHO classification separates prepubertal-type teratomas and YSTs (non-GCNIS derived) from postpubertal types that are GCNIS-associated [7].

Although most molecular knowledge has been generated from testicular tumors, smaller studies have also examined ovarian and mediastinal GCTs [16,17,18,19]. These investigations indicate that some genetic alterations are shared across malignant GCTs regardless of primary site, while others vary according to site of origin or histologic subtype [2]. Moreover, even within a single histologic subtype, distinct molecular subgroups can be defined based on differing genetic profiles [20]. Such findings have led to developmental models of classification, initially dividing GCTs into five types and later expanded into seven, underscoring the growing influence of molecular biology on tumor taxonomy [1,15].

Despite these advances, clinical research on malignant GCTs remains limited, especially with regard to metastatic patterns [21]. This gap can be attributed to three main factors: (1) malignant GCTs are rare, (2) most cases are diagnosed at an early organ-confined stage, and (3) their generally favorable outcome with modern therapies has lowered their research priority compared with other malignancies [10,11,22].

Nevertheless, a subset of patients relapse or experience poor outcomes [7,21,23]. Because these tumors primarily affect young individuals, long-term surveillance is essential, and follow-up strategies must be effective, economical, and minimally burdensome. Studying metastatic patterns may help address this need by providing clinically relevant guidance for patient care. In addition, given the diversity of primary sites, histologic subtypes, and molecular features of malignant GCTs, such analyses may also yield important insights into the mechanisms of tumor dissemination.

Accordingly, this study aims to compare the metastatic patterns of malignant GCTs arising in the testis, ovary, and mediastinum, with particular attention to their histologic subtype and site of origin. Because malignant GCTs are rare, the analysis was conducted using data from the Surveillance, Epidemiology, and End Results (SEER) program, a large U.S. population-based cancer registry maintained by the National Cancer Institute that collects comprehensive data on cancer incidence, patient demographics, and tumor characteristics across multiple regions.

## 2. Materials and Methods

### 2.1. Data Source and Patient Selection

Patients diagnosed with malignant GCTs between 2010 and 2022 were selected from the SEER program using the SEER*Stat software version 9.0.41.4 [24]. For this study, the database “Incidence—SEER Research Data, 17 Registries, Nov 2024 Sub (2000–2022)” was used [25].

Case selection was performed through the Case Listing Session by applying specific site and histologic diagnosis restrictions within the Site and Morphology category. For the primary site, the variable “Site recode ICD-O-3/WHO 2008” was used, and cases were included if their values corresponded to “Trachea, Mediastinum and Other Respiratory Organs”, “Ovary”, and “Testis”. For the histologic diagnosis, the variable “ICD-O-3 Hist/behav, malignant” was applied, and cases were selected if their values matched the following codes: “9060/3: Dysgerminoma”, “9061/3: Seminoma, NOS”, “9070/3: Embryonal carcinoma, NOS”, “9071/3: Yolk sac tumor”, “9080/3: Teratoma, malignant, NOS”, “9085/3: Mixed germ cell tumor”, and “9100/3: Choriocarcinoma, NOS”.

For each selected case, data were extracted for statistical analysis. Extracted variables included patient ID, sex, age at diagnosis, histologic diagnosis, information on metastatic status at diagnosis (bone, brain, liver, lung, distant lymph nodes, and other organs), as well as N stage and M stage.

Mixed GCTs were analyzed as a distinct category because the SEER database does not provide information on the relative proportions or dominant components of individual histologic elements. Therefore, component-specific or dominant-type analyses could not be performed.

For transparency and reproducibility, the detailed SEER*Stat extraction procedure, including all selection filters, variable definitions, and exported fields, is described in Supplementary Materials Table S3. A completed STROBE checklist (cohort studies) is also provided as Supplementary Material.

### 2.2. Data Processing and Analysis

In the SEER database, information on metastases to bone, brain, liver, and lung has been available since 2010, whereas data on metastases to other organs and distant lymph nodes have only been collected since 2016. To ensure consistency across the study period, the presence of distant metastasis in each case was determined using the data on the M stage, and the distant organ metastasis rate was calculated accordingly. Similarly, lymph node involvement was evaluated using the data on N stage, and lymph node metastasis rates were derived on this basis.

To verify the robustness of the results, a sensitivity analysis restricted to cases diagnosed between 2016 and 2022, when complete information on all metastatic sites was available, was performed (Table S1). The findings were consistent with those from the full cohort (2010–2022). It should also be noted that SEER records metastatic status only at the time of diagnosis, so the present analysis reflects metastatic presentation rather than subsequent progression.

Cases were then stratified by primary site and histologic diagnosis. In addition, they were subdivided into prepubertal and postpubertal categories. These categories follow the conventional developmental classification of germ cell tumors and do not refer to physiological pubertal status. Previous studies have demonstrated that these two groups differ in their molecular and genetic characteristics, making it necessary to distinguish between them [1]. Although no single standard age cutoff exists, previous studies have indicated thresholds within the 5–8 year range, including approximately 5 years [26], 6 years [1], and 8 years [18].

In the present study, both 5- and 8-year thresholds were preliminarily evaluated, and no substantial difference was observed between the two (Table S2). To minimize potential inclusion of prepubertal-type tumors and ensure comparability within the postpubertal-type group, an 8-year cutoff was applied for the final analysis.

Next, metastatic patterns were compared across groups defined by primary site and histologic diagnosis. Statistically significant differences in the overall distant organ metastasis rate were observed among these groups. In such situations, calculating organ-specific metastasis rates (e.g., for bone, brain, liver, or lung) in the same manner as the overall metastasis rate can introduce bias. The overall metastasis rate, defined as the percentage of patients with metastases among all patients, is not suitable for comparing organ-specific metastatic tendencies, because tumors with higher overall rates will automatically appear to have higher organ-specific rates, and vice versa. This leads to statistical distortion and prevents accurate comparison between tumors with different overall rates.

To address this issue, organ-specific metastasis was instead expressed as the proportion of patients with metastasis to a given organ among those who had any metastasis. This adjusted measure, referred to as the organotropic metastasis rate, has been applied in previous studies [27,28] and was also adopted in the present study.

### 2.3. Statistical Analysis

Comparisons of categorical variables, including the presence or absence of metastases, were carried out using either Pearson’s chi-square test or Fisher’s exact test, depending on appropriateness. All statistical analyses were descriptive and unadjusted. Formal sample size estimation was not feasible due to the small number of cases in several subgroups, particularly those with mediastinal or ovarian primaries, and these comparisons were interpreted descriptively. In line with the descriptive aim of this study rather than the identification of independent predictors, multivariable regression modeling was not performed. All statistical computations were conducted with SPSS software (version 17.0; SPSS Inc., Chicago, IL, USA), and graphical analyses and visualization were performed using OriginPro (version 2025; OriginLab, Northampton, MA, USA).

## 3. Results

### 3.1. Patient Characteristics

Between 2010 and 2022, a total of 32,015 malignant GCT cases were identified, including 29,768 (93.0%) testicular, 1785 (5.6%) ovarian, and 462 (1.4%) mediastinal tumors. As expected, all testicular tumors occurred in males, whereas all ovarian tumors occurred in females. In mediastinal tumors, sex distribution varied by age at diagnosis, with females predominating among patients younger than 8 years (male-to-female ratio 1:3.5), while males predominated in older patients (28.6:1).

For analytic clarity, cases were stratified by age at diagnosis (<8 years vs. ≥8 years). In the younger group (*n* = 195), there were 130 testicular, 47 ovarian, and 18 mediastinal tumors. The distribution of histologic subtypes, together with the rates of lymph node and distant metastases, is summarized in Table 1. Overall, lymph node metastasis was observed in 10 cases (5.1%), while distant metastasis was observed in 13 cases (7.0%). Lymph node metastasis rates did not differ significantly across primary sites. However, distant metastasis rates were significantly higher in mediastinal tumors (21.4%, 3/14) compared with testicular tumors (4.0%, 5/126; *p* = 0.033). Although mediastinal tumors also had higher rates than ovarian tumors (10.6%, 5/47), the difference did not reach statistical significance (*p* = 0.369). Because of the limited number of cases, histology-specific analysis could not be performed in this group.

**Figure 1:**
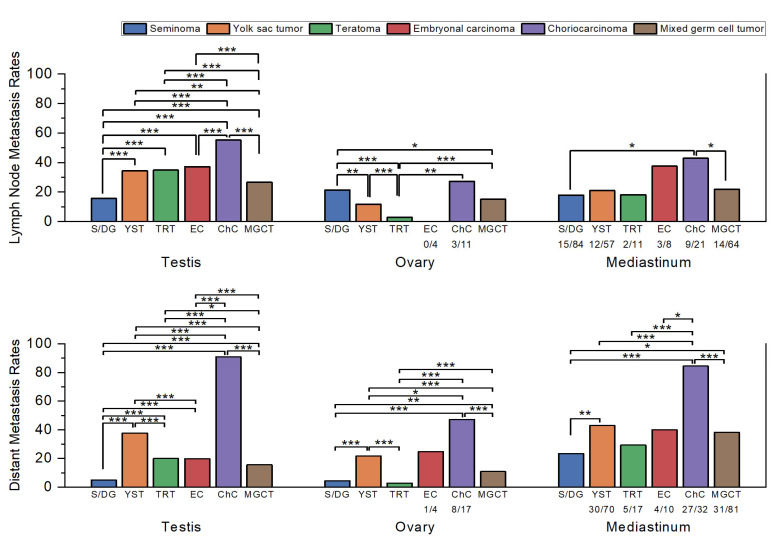
Comparison of the lymph node and distant metastasis rates by histologic subtype in each primary site. Sample sizes (*n*/*N*) are shown below each bar for subgroups with fewer than 100 cases. Statistically significant comparisons are marked with asterisks (*, *p* ≤ 0.05; **, *p* ≤ 0.01; ***, *p* ≤ 0.001). Statistical comparisons were performed using Pearson’s chi-square test or Fisher’s exact test, as appropriate, and *p*-values were interpreted descriptively without formal correction for multiple testing. Abbreviations: S/DG, seminoma/dysgerminoma; YST, yolk sac tumor; TRT, teratoma; EC, embryonal carcinoma; ChC, choriocarcinoma; MGCT, mixed germ cell tumor.

In the ≥8-year group (*n* = 31,820), 29,638 (93.1%) were testicular, 1738 (5.5%) were ovarian, and 444 (1.4%) were mediastinal tumors. Their histologic distribution and metastatic rates are presented in Table 1 and Figure 1. Ovarian tumors showed significantly lower lymph node metastasis rates (11.8%) compared with testicular (21.6%) and mediastinal tumors (22.4%; both *p* < 0.001), with no significant difference between testicular and mediastinal tumors (*p* = 0.761). A similar pattern was observed for distant metastasis. Ovarian tumors had the lowest rate (8.1%), significantly lower than both testicular (10.8%) and mediastinal tumors (38.8%; both *p* < 0.001). In contrast to lymph node metastasis, mediastinal tumors exhibited significantly higher distant metastasis rates compared with testicular tumors (*p* < 0.001).

### 3.2. Comparison of Lymph Node and Distant Metastasis Rates by Histologic Subtype in Each Primary Site

When stratified by histologic subtype, clear differences in lymph node and distant metastasis rates were observed (Table 1 and Figure 1).

In testicular GCTs, seminoma had the lowest lymph node metastasis rate (15.7%, 2418/15,448), significantly lower than all other subtypes (*p* < 0.001). Mixed GCTs (26.7%, 2309/8647) showed higher rates than seminoma (*p* < 0.001) but remained significantly lower than YST (*p* = 0.004) and the other subtypes (*p* < 0.001). YST (34.4%, 99/288), teratoma (34.9%, 149/427), and EC (37.2%, 777/2089) did not differ significantly, while CHC (55.2%, 116/210) showed the highest rate, significantly greater than all others (*p* < 0.001).

For distant metastasis, seminoma (5.0%, 842/16,831) again had the lowest rate (*p* < 0.001 vs. all). Mixed GCT (15.6%, 1463/9353) occupied an intermediate position, significantly higher than seminoma (*p* < 0.001) but lower than teratoma (*p* = 0.01) and the other subtypes (*p* < 0.001). EC (19.9%, 453/2277) and teratoma (20.1%, 94/468) were comparable (*p* = 0.925). YST (37.9%, 128/338) was significantly higher than all except CHC (*p* < 0.001), while CHC (90.8%, 217/239) had the highest rate overall (*p* < 0.001).

In ovarian GCTs, EC was excluded from analysis due to the limited number of cases (*n* = 4). Teratoma showed the lowest lymph node metastasis rate (2.9%, 18/620), significantly lower than all other groups (*p* < 0.001 vs. most; *p* = 0.004 vs. CHC). YST (11.8%, 31/262) and mixed GCT (15.2%, 39/257) were not significantly different, whereas dysgerminoma (21.5%, 100/465) was significantly higher than both YST (*p* = 0.001) and mixed GCT (*p* = 0.039). CHC (27.3%, 3/11) showed the numerically highest rate, though statistical significance was not reached, likely because of the small number of cases.

For distant metastasis, teratoma (2.9%, 19/662) again had the lowest rate, significantly lower than all groups except dysgerminoma (*p* = 0.156 vs. dysgerminoma; *p* < 0.001 vs. others). Dysgerminoma (4.4%, 22/497) was lower than mixed GCT (10.9%, 29/267; *p* = 0.001) and the higher-rate subtypes. Mixed GCT represented an intermediate group, significantly lower than YST and CHC (both *p* < 0.001). YST (21.8%, 62/284) demonstrated a high rate, while CHC (47.1%, 8/17) was the highest, significantly greater than all others (*p* = 0.033 vs. YST; *p* < 0.001 vs. others).

In mediastinal GCTs, seminoma had the lowest lymph node metastasis rate (17.9%, 15/84), followed by teratoma (18.2%, 2/11), YST (21.1%, 12/57), and mixed GCT (21.9%, 14/64). EC (37.5%, 3/8) and CHC (42.9%, 9/21) were higher, with significant differences only between seminoma and CHC (*p* = 0.015) and between mixed GCT and CHC (*p* = 0.03).

For distant metastasis, seminoma (23.5%, 24/102) again had the lowest rate, while CHC (84.4%, 27/32) was the highest. Intermediate rates were observed in teratoma (29.4%, 5/17), mixed GCT (38.3%, 31/81), EC (40.0%, 4/10), and YST (42.9%, 30/70). Significant differences were identified between seminoma and mixed GCT (*p* = 0.031) and between CHC and all other groups (*p* = 0.011 vs. EC; *p* < 0.001 vs. others). These findings should be interpreted with caution, as the lack of statistical significance in many comparisons is likely attributable to the limited number of mediastinal cases.

### 3.3. Comparison of Organotropic Metastasis Rates by Histologic Subtype in Each Primary Site

In accordance with the Methods Section, organotropic metastasis rates were used to compare metastatic patterns across histologic subtypes, thereby avoiding bias introduced by overall metastasis rates.

The organotropic metastasis rate was defined as the proportion of patients with metastasis to a specific distant organ (e.g., lung, liver, brain, bone, or other sites) among those who had any distant metastasis.

This approach allows an accurate comparison of organotropic metastatic patterns between tumor groups with differing overall distant metastatic rates.

#### 3.3.1. Testicular GCTs

**Figure 2:**
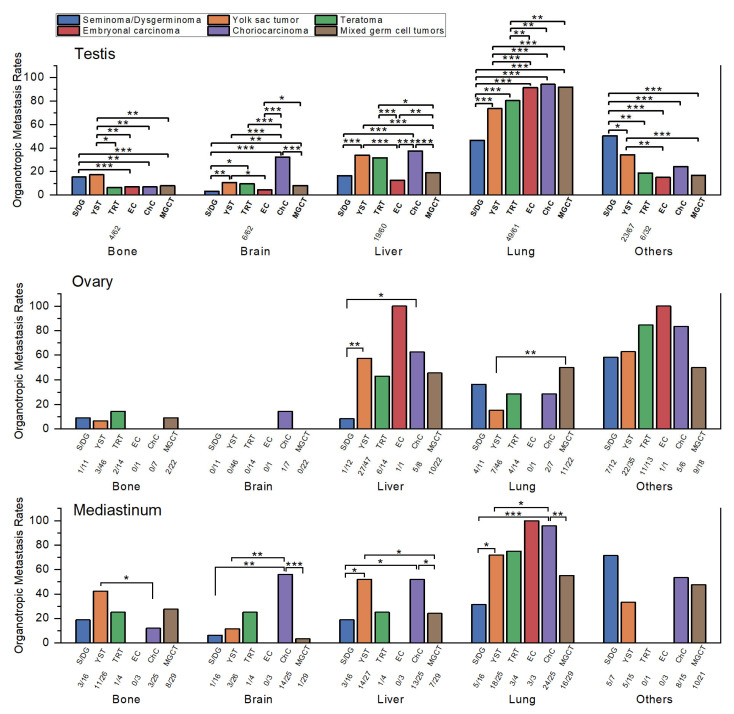
Comparison of organotropic metastasis rates of malignant germ cell tumors by histologic subtype within each primary site. Sample sizes (*n*/*N*) are shown below each bar for subgroups with fewer than 100 cases. Statistically significant comparisons are marked with asterisks (*, *p* ≤ 0.05; **, *p* ≤ 0.01; ***, *p* ≤ 0.001). Statistical comparisons were performed using Pearson’s chi-square test or Fisher’s exact test, as appropriate, and *p*-values were interpreted descriptively without formal correction for multiple testing. Abbreviations: S/DG, seminoma/dysgerminoma; YST, yolk sac tumor; TRT, teratoma; EC, embryonal carcinoma; ChC, choriocarcinoma; MGCT, mixed germ cell tumor.

Clear differences were observed among subtypes (Table 2 and Figure 2).

Bone metastasis was most frequent in YST (17.3%, 18/104) and seminoma (15.3%, 60/392), both significantly higher than mixed GCT (7.8%, 92/1182), EC (7.0%, 24/344), and CHC (6.9%, 14/204; all *p* ≤ 0.004). Teratoma had the lowest rate (6.5%, 4/62), significantly lower than YST (*p* = 0.046) but not different from seminoma (*p* = 0.063), likely due to the small number of teratoma cases.

Brain metastasis was highest in CHC (32.5%, 67/206; *p* < 0.001 vs. all). YST (10.7%, 11/103), teratoma (9.7%, 6/62), and mixed GCT (8.2%, 97/1180) followed, with no significant differences among them. EC (4.4%, 15/342) and seminoma (3.3%, 13/393) showed the lowest rates, both significantly lower than YST and mixed GCT (*p* ≤ 0.017).

Liver metastasis was most frequent in CHC (37.6%, 77/205), YST (34.0%, 36/106), and teratoma (31.7%, 19/60), which did not differ significantly from each other but were all higher than mixed GCT (19.0%, 224/1181), seminoma (16.6%, 65/392), and EC (12.5%, 43/344; all *p* ≤ 0.016). The comparison between seminoma and teratoma demonstrated borderline significance (*p* = 0.05), possibly reflecting the limited number of teratoma cases. Among the lower-rate subtypes, mixed GCT had a significantly higher rate than EC (*p* = 0.005) but did not differ significantly from seminoma. Seminoma and EC also showed no significant difference (*p* = 0.118).

Lung metastasis predominated across all subtypes. CHC (94.2%, 194/206), mixed GCT (91.9%, 1093/1189), and EC (91.4%, 317/347) were highest, without significant differences among them. Teratoma (80.3%, 49/61) and YST (73.6%, 78/106) were significantly lower than the three highest groups (*p* ≤ 0.009) but not different from each other. Seminoma had the lowest rate (46.7%, 184/394), significantly lower than all others (*p* < 0.001).

Other organ metastasis was most frequent in seminoma (50.4%, 143/284), followed by YST (34.3%, 23/67), CHC (24.1%, 26/108), teratoma (18.8%, 6/32), mixed GCT (16.9%, 120/711), and EC (15.1%, 31/205). Seminoma was significantly higher than all other groups (*p* ≤ 0.018). YST was significantly higher than mixed GCT and EC, but not different from CHC. The remaining subtypes clustered at relatively low levels without meaningful differences. Because SEER began recording “other organ” metastases only recently, small sample sizes likely limited statistical power.

#### 3.3.2. Ovarian GCTs

EC was excluded from analysis due to the very small sample size (*n* = 4). Because of limited case numbers, most comparisons were not statistically significant, though several trends were observed (Table 2 and Figure 2).

Bone metastasis was highest in teratoma (14.3%, 2/14), followed by dysgerminoma (9.1%, 1/11), mixed GCT (9.1%, 2/22), YST (6.5%, 3/46), and CHC (0%, 0/7). None of these differences reached statistical significance (all *p* > 0.5).

Brain metastasis was also uncommon, with CHC (14.3%, 1/7) being numerically higher than other subtypes (mostly 0%), though not statistically significant (*p* = 0.389 vs. dysgerminoma).

Liver metastasis was most frequent in CHC (62.5%, 5/8), YST (57.4%, 27/47), mixed GCT (45.5%, 10/22), and teratoma (42.9%, 6/14), all significantly higher than dysgerminoma (8.3%, 1/12; *p* ≤ 0.018).

Lung metastasis was highest in mixed GCT (50.0%, 11/22), followed by dysgerminoma (36.4%, 4/11), teratoma (28.6%, 4/14), CHC (28.6%, 2/7), and YST (15.2%, 7/46). The only significant difference was between mixed GCT and YST (*p* = 0.002).

Other organ metastasis was highest in teratoma (84.6%, 11/13) and CHC (83.3%, 5/6), followed by YST (62.9%, 22/35), dysgerminoma (58.3%, 7/12), and mixed GCT (50.0%, 9/18). Despite the apparent numerical gradient, no differences were statistically significant, likely due to limited case numbers.

#### 3.3.3. Mediastinal GCTs

As in ovarian tumors, small sample sizes limited statistical analysis. EC and teratoma were excluded from comparisons because of very few cases. Despite these constraints, certain organotropic patterns were evident (Table 2 and Figure 2).

Bone metastasis was most frequent in YST (42.3%, 11/26), followed by mixed GCT (27.6%, 8/29), seminoma (18.8%, 3/16), and CHC (12.0%, 3/25). YST was significantly higher than CHC (*p* = 0.015), while other comparisons did not reach statistical significance.

Brain metastasis was highest in CHC (56.0%, 14/25), which was significantly greater than YST (11.5%, 3/26), seminoma (6.3%, 1/16), and mixed GCT (3.4%, 1/29; all *p* ≤ 0.001).

Liver metastasis was also most frequent in YST (51.9%, 14/27) and CHC (52.0%, 13/25), which were comparable to one another and significantly higher than seminoma (18.8%, 3/16) and mixed GCT (24.1%, 7/29; *p* ≤ 0.035).

Lung metastasis was the dominant pattern across mediastinal tumors. CHC had the highest rate (96.0%, 24/25), significantly greater than YST (72.0%, 18/25), mixed GCT (55.2%, 16/29), and seminoma (31.3%, 5/16; *p* ≤ 0.049).

Other organ metastasis was most frequent in seminoma (71.4%, 5/7), followed by CHC (53.3%, 8/15), mixed GCT (47.6%, 10/21), and YST (33.3%, 5/15). None of these differences reached statistical significance, likely due to the small number of cases.

### 3.4. Comparison of Metastatic Patterns Across Primary Sites

**Figure 3:**
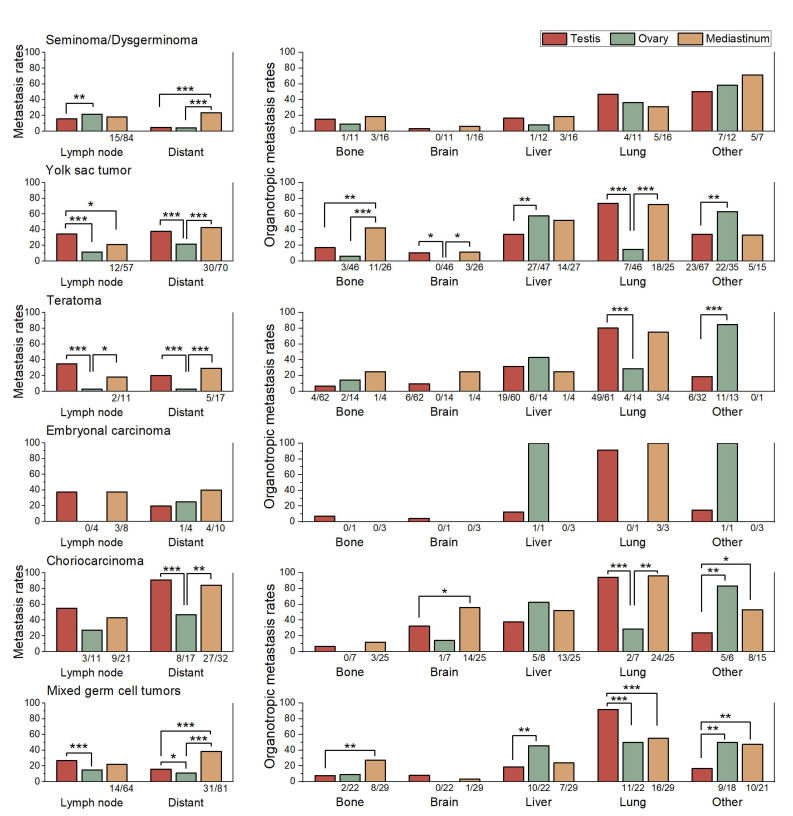
Comparison of lymph node metastasis rates, distant metastasis rates, and organotropic metastasis rates to bone, brain, liver, lung, and other organs across primary sites (testis, ovary, and mediastinum) within each histologic subtype. Sample sizes (*n*/*N*) are shown below each bar for subgroups with fewer than 100 cases. Statistically significant comparisons are marked with asterisks (*, *p* ≤ 0.05; **, *p* ≤ 0.01; ***, *p* ≤ 0.001). Statistical comparisons were performed using Pearson’s chi-square test or Fisher’s exact test, as appropriate.

When lymph node and distant metastasis rates were compared across primary sites, several consistent trends were observed (Figure 3). Ovarian GCTs generally exhibited lower lymph node metastasis rates than testicular and mediastinal tumors, whereas mediastinal GCTs tended to show higher distant metastasis rates across most histologic subtypes. Among these, significant contrasts were identified in two subtypes: seminoma (23.5% compared with 5.0%) and mixed GCT (38.3% compared with 15.6%), both showing higher distant metastasis rates in mediastinal than in testicular tumors.

Choriocarcinoma (CHC) exhibited a characteristically high brain organotropic metastasis rate across all primary sites, with 56% in mediastinal and 32.5% in testicular tumors, indicating that its strong predilection for brain metastasis is an inherent feature rather than site dependent and consistent with its known aggressive clinical behavior.

Comparison of organotropic metastasis rates by primary site revealed several observable trends (Table 2 and Figure 3). However, for EC, the very limited number of cases precluded meaningful statistical comparisons.

For seminoma/dysgerminoma, the organotropic metastasis pattern was generally consistent across different primary sites. In contrast, the other subtypes exhibited clear variations in organotropic metastasis rates depending on the primary site.

In YSTs, mediastinal tumors were characterized by a markedly greater tendency for bone metastasis compared with testicular and ovarian tumors (*p* ≤ 0.006). Testicular tumors, in turn, showed a lower propensity for liver metastasis than ovarian tumors (*p* = 0.006). Ovarian tumors demonstrated a distinctly lower likelihood of lung metastasis than both testicular and mediastinal tumors (*p* ≤ 0.001), while exhibiting a higher frequency of metastasis to other organs compared with testicular tumors (*p* = 0.006).

In teratomas, ovarian tumors exhibited a markedly higher propensity for metastasis to other organs compared with testicular tumors (84.6% vs. 18.8%; *p* < 0.001). Conversely, the lung organotropic metastasis rate was significantly lower in ovarian tumors than in testicular (28.6% vs. 80.3%; *p* < 0.001) and mediastinal tumors (75.0%), although the latter comparison did not reach statistical significance because of the limited case number.

In CHC, mediastinal tumors exhibited a significantly higher brain organotropic metastasis rate than testicular tumors (56.0% vs. 32.5%; *p* = 0.020). The organotropic metastasis rate to other organs was significantly lower in testicular tumors (24.1%) compared with mediastinal (53.3%) and ovarian tumors (83.3%; *p* ≤ 0.029). And ovarian tumors showed a markedly lower lung organotropic metastasis rate (28.6%) than testicular (94.2%) and mediastinal tumors (96.0%; *p* ≤ 0.001).

In mixed GCTs, testicular tumors showed the highest organotropic metastasis rate to the lung (91.9%), which was significantly greater than ovarian (50.0%) and mediastinal tumors (55.2%; both *p* ≤ 0.001). By contrast, the organotropic metastasis rate to other organs was lowest in testicular tumors (16.9%) compared with ovarian (50.0%) and mediastinal tumors (47.6%; *p* ≤ 0.002). Ovarian tumors demonstrated a significantly higher organotropic metastasis rate to the liver (45.5%) than testicular tumors (19.0%; *p* = 0.005). Mediastinal tumors showed the highest organotropic metastasis rate to bone (27.6%), which exceeded testicular (7.8%) and ovarian tumors (9.1%), though these differences did not reach statistical significance.

## 4. Discussion

Malignant germ cell tumors (GCTs) are clinically significant malignancies, yet their rarity and histologic heterogeneity have limited research compared with more common cancers. Using a population-based database, the present study compared overall metastasis rates as well as organotropic metastatic patterns across different primary sites and histologic subtypes, an area rarely explored in previous research.

The patient cohort in this study reflected the well-established epidemiologic distribution of GCTs, with testicular tumors comprising the vast majority, ovarian tumors being less frequent, and mediastinal tumors rare [2]. Sex distribution aligned with expectations in gonadal tumors, whereas mediastinal tumors showed female predominance in early childhood and male predominance thereafter, a pattern consistent with prior studies [9]. Histologically, seminoma was more common than non-seminoma in males, whereas the opposite trend was seen in females, also consistent with previous reports [2]. These patterns underscore the representativeness of our cohort and provide a robust basis for subsequent comparisons of metastatic behavior.

Overall lymph node and distant metastasis rates appeared to differ by both histologic subtype and primary site. Seminoma and dysgerminoma generally tended to show lower distant metastasis rates than non-seminomatous or non-dysgerminomatous GCTs arising from the same primary site. Likewise, lymph node metastasis rates were typically lower in testicular and mediastinal seminomas than in their non-seminomatous counterparts. Interestingly, however, dysgerminoma showed a higher frequency of lymph node metastasis not only compared with testicular or mediastinal seminoma but also with most ovarian non-dysgerminomatous GCTs, except for choriocarcinoma. This distinctive pattern has been reported in previous studies [29,30].

The distinctive nature of ovarian GCTs was also evident when comparing the metastatic behavior of non-seminomatous or non-dysgerminomatous GCTs. When differences by primary site were examined, mediastinal tumors tended to show the highest distant metastasis rates across most histologic subtypes, which was largely expected given that the mediastinal primary site has long been recognized as a poor prognostic factor in non-seminomatous GCTs [31]. However, the finding that ovarian non-dysgerminomatous GCTs exhibited distant metastasis rates comparable to or even lower than those of testicular tumors across all histologic subtypes was unexpected and intriguing, since ovarian non-dysgerminomatous GCTs are generally regarded as having a poorer prognosis than testicular tumors [20,32].

The underlying reason for this discrepancy remains unclear, but several potential explanations can be considered.

One possible explanation is that distant metastasis itself may have a relatively limited impact on prognosis. This assumption might seem reasonable given that, for lymph node metastasis, its prognostic significance remains controversial and is often considered limited because of the high chemosensitivity of GCTs [29,33,34]. However, unlike lymph node metastasis, distant metastasis has been repeatedly reported as a significant adverse prognostic factor in multiple studies [6,35,36]. Therefore, the hypothesis that distant metastasis exerts only a limited effect on prognosis is unlikely to be correct.

If distant metastasis does contribute to adverse outcomes, the poorer prognosis observed in ovarian GCTs must stem from other factors.

One possibility is that ovarian GCTs have worse outcomes because they are far less studied and consequently less well treated than testicular GCTs. This notion is supported by the fact that current therapeutic approaches for ovarian GCTs are largely extrapolated from data on testicular GCTs, and standardized treatment protocols remain underdeveloped [32,34,37]. In particular, for patients with recurrent disease, no established standard therapy currently exists, which likely contributes to their markedly poorer prognosis [34].

If this hypothesis is correct, ovarian GCTs may represent a disease entity with the potential for substantially improved outcomes through the development of optimized, evidence-based treatment strategies. Therefore, further research focusing on their unique clinical and biological characteristics is warranted.

As mentioned above, the high distant metastasis rates observed in mediastinal germ cell tumors in this study are consistent with previous reports identifying mediastinal origin as an adverse prognostic factor. However, the underlying reason why mediastinal GCTs show poorer outcomes than their testicular counterparts remains unclear and lies beyond the scope of this study. It has been suggested, however, that at least part of this difference may be attributable to delayed diagnosis compared with testicular GCTs [9].

Beyond overall metastasis rates, differences in organotropic metastatic patterns were also evident across histologic subtypes and primary sites. Whereas overall rates reflect the general metastatic potential of each tumor type, organotropic patterns provide deeper insight into the preferred routes and destinations of dissemination. Analysis of these patterns suggested distinct site- and subtype-specific tendencies, underscoring the biological diversity of malignant GCTs. Although statistical analysis was limited by the small number of cases, particularly among ovarian and mediastinal tumors, certain trends could still be identified, and these patterns alone provided several notable and meaningful observations.

Among the histologic subtypes, CHC consistently exhibited the most aggressive metastatic behavior, with a strong tropism for the lung and liver and a particularly high propensity for brain metastasis, especially in mediastinal and testicular tumors. These findings are consistent with its well-known association with poor clinical outcomes.

YST also demonstrated frequent metastases to the liver and bone, and to a lesser extent the lung, with some variation depending on the primary site.

Mixed GCTs showed intermediate patterns, with lung metastasis being common but variable involvement of other organs, reflecting differences in their histologic composition.

Seminoma and dysgerminoma demonstrated lower organotropic metastasis rates to the brain, liver, and lung, while showing a relatively higher rate of metastasis to other organs.

When organotropic metastasis rates were compared by primary site within each histologic subtype, seminoma and dysgerminoma showed relatively similar patterns regardless of tumor origin. Because the number of ovarian dysgerminomas and mediastinal seminomas in this study was small, it is difficult to determine whether this reflects a true absence of statistical difference or simply the limitation of small sample size. Nevertheless, the overall tendency appeared comparable across primary sites.

In contrast, non-seminomatous and non-dysgerminomatous GCTs demonstrated clear differences in organotropic metastasis patterns depending on the primary site. Mediastinal non-seminomatous GCTs showed a clear tendency toward more frequent and extensive distant metastases than their testicular or ovarian counterparts. In particular, mediastinal YSTs exhibited a markedly higher bone organotropic metastasis rate, while mediastinal CHCs showed a notably greater tendency for brain metastasis compared with tumors of gonadal origin. These findings are consistent with previous reports identifying mediastinal origin as an adverse prognostic factor.

Ovarian non-dysgerminomatous GCTs showed distinct organotropic features. Across several subtypes, ovarian non-dysgerminomatous GCTs consistently exhibited lower lung organotropic metastasis rates than both testicular and mediastinal non-seminomatous GCTs, while showing relatively higher organotropic metastasis rates to other organs. These findings suggest that metastatic behavior in non-dysgerminomatous GCTs is not determined solely by histologic subtype but may also be influenced by additional factors.

This distinctive organotropic pattern observed in ovarian non-dysgerminomatous GCTs raises important questions regarding its underlying mechanisms. Several possible explanations can be proposed. One is that the anatomic characteristics of the ovary itself may contribute to this pattern. Another is that hormonal or other sex-related environmental factors may play a role, given that testicular GCTs occur exclusively in males and mediastinal GCTs predominantly affect males. Finally, molecular and genetic differences between ovarian non-dysgerminomatous GCTs and testicular non-seminomatous GCTs may also account for these distinct metastatic tendencies.

However, none of these hypotheses can fully account for this phenomenon. If the distinctive pattern were primarily attributable to the anatomic characteristics of the ovary or to sex-related factors, a similar distribution of metastasis would be expected not only in non-dysgerminomatous GCTs but also in dysgerminomas. This leaves the possibility that the pattern may instead reflect underlying molecular and genetic characteristics specific to ovarian non-dysgerminomatous GCTs.

Indeed, several studies have reported that at least some subtypes of ovarian non-dysgerminomatous GCTs exhibit molecular and genetic profiles distinct from their testicular and mediastinal counterparts [16]. For example, in malignant teratoma, the 12p alteration commonly observed in testicular malignant teratomas is typically absent in pure immature teratomas of ovarian origin [20].

However, such molecular differences have been identified within specific histologic subtypes rather than across all ovarian non-dysgerminomatous GCTs as a group. Therefore, if molecular alterations underlie differences in metastatic behavior, their effects are likely confined to individual subtypes rather than shared universally among ovarian non-dysgerminomatous GCTs.

To date, no molecular or genetic alteration has been identified that is consistently present in ovarian non-dysgerminomatous GCTs but absent in non-seminomatous GCTs of other primary sites. Consequently, the precise mechanism underlying these unique organotropic features remains uncertain and warrants further investigation.

The present study provides a comprehensive overview of metastatic tendencies in malignant GCTs; however, several limitations should be acknowledged when interpreting these findings.

First, although the SEER database offers extensive population-level coverage, data on metastatic sites have been collected only in recent years. As a result, the number of ovarian and mediastinal cases, particularly those with distant metastases, was limited, which inevitably reduced the statistical power for certain subgroup analyses.

Because of these small subgroup sizes and the descriptive nature of the analysis, multivariable modeling was not performed, and potential confounding by factors such as age, sex, histology, or primary site could not be fully controlled.

Nevertheless, these subgroups were retained in the analysis because their inclusion was considered informative for descriptive comparison. To enhance transparency, sample sizes for smaller subgroups are now indicated in Figure 1, Figure 2 and Figure 3, and no inferential conclusions were drawn from underpowered comparisons.

Second, the SEER database records only the metastatic status at the time of initial diagnosis and does not include information on disease progression or subsequent metastatic spread. Therefore, temporal changes in metastatic patterns could not be evaluated.

Third, histologic classification could not be independently verified by reviewing pathology slides, leaving the possibility of misclassification inherent to registry-based studies.

Fourth, molecular profiles were unavailable, making it difficult to distinguish between prepubertal and postpubertal forms of YST, which are known to differ in their pathogenesis.

Finally, for mixed GCTs, the SEER data do not specify the individual histologic components, which precluded more detailed analyses of how specific elements contribute to metastatic patterns. Consequently, potential organotropic patterns driven by dominant histologic elements could not be separately assessed. Mixed GCTs were therefore retained as a distinct category to preserve overall case numbers, especially for rare primary sites. Although this approach has inherent heterogeneity, mixed GCTs represent a relatively common subtype, and including them provides clinically informative, albeit approximate, insight into their metastatic behavior.

Despite these limitations, the strength of this study lies in its large, population-based design encompassing multiple primary sites and histologic subtypes. This comprehensive approach provides a rare opportunity to examine metastatic patterns in malignant GCTs from a broad comparative perspective that single-institution series cannot easily achieve.

## 5. Conclusions

This study provides a population-based overview of metastatic patterns in malignant GCTs, suggesting that both histologic subtype and primary site may influence the routes of tumor dissemination. By introducing organotropic metastasis rate as an analytical measure, it provides a framework for descriptively characterizing metastatic tendencies that may inform future research and clinical surveillance. Future studies integrating molecular and clinical data will be important to further clarify the biological mechanisms underlying these differences and to support the development of site-specific management approaches for patients with malignant GCTs.

## Figures and Tables

**Table 1:** Overall lymph node and distant metastasis rates of malignant germ cell tumors stratified by age group, primary site, and histologic subtype.

	Age at Diagnosis < 8			Age at Diagnosis ≥ 8		
Diagnosis	*N*	LNM (%) (*n*/*N*)	DM (%) (*n*/*N*)	*N*	LNM (%) (*n*/*N*)	DM (%) (*n*/*N*)
Testicular GCTs						
Seminoma	1	0% (0/0)	100% (1/1)	16,908	15.7% (2418/15,448)	5.0% (842/16,831)
YST	94	6.0% (5/83)	4.3% (4/92)	339	34.4% (99/288)	37.9% (128/338)
Teratoma	25	0% (0/22)	0% (0/24)	473	34.9% (149/427)	20.1% (94/468)
EC	3	33.3% (1/3)	0% (0/3)	2281	37.2% (777/2089)	19.9% (453/2277)
CHC	0	0% (0/0)	0% (0/0)	239	55.2% (116/210)	90.8% (217/239)
Mixed GCT	7	0% (0/5)	0% (0/6)	9398	26.7% (2309/8647)	15.6% (1463/9353)
Total	130	5.3% (6/113)	4.0% (5/126)	29,638	21.6% (5868/27,109)	10.8% (3197/29,506)
Ovarian GCTs						
Dysgerminoma	4	25.0% (1/4)	0% (0/4)	497	21.5% (100/465)	4.4% (22/497)
YST	5	25.0% (1/4)	40.0% (2/5)	284	11.8% (31/262)	21.8% (62/284)
Teratoma	25	0% (0/24)	8% (2/25)	667	2.9% (18/620)	2.9% (19/662)
EC	1	0% (0/1)	0% (0/1)	4	0% (0/4)	25.0% (1/4)
CHC	0	0% (0/0)	0% (0/0)	17	27.3% (3/11)	47.1% (8/17)
Mixed GCT	12	9.1% (1/11)	8.3% (1/12)	269	15.2% (39/257)	10.9% (29/267)
Total	47	6.8% (3/44)	10.6% (5/47)	1738	11.8% (191/1619)	8.1% (141/1731)
Mediastinal GCTs						
Seminoma	0	0% (0/0)	0% (0/0)	156	17.9% (15/84)	23.5% (24/102)
YST	4	25.0% (1/4)	75.0% (3/4)	99	21.1% (12/57)	42.9% (30/70)
Teratoma	13	0% (0/8)	0% (0/9)	24	18.2% (2/11)	29.4% (5/17)
EC	0	0% (0/0)	0% (0/0)	15	37.5% (3/8)	40.0% (4/10)
CHC	0	0% (0/0)	0% (0/0)	34	42.9% (9/21)	84.4% (27/32)
Mixed GCT	1	0% (0/1)	0% (0/1)	116	21.9% (14/64)	38.3% (31/81)
Total	18	7.7% (1/13)	21.4% (3/14)	444	22.4% (55/245)	38.8% (121/312)

LNM, lymph node metastasis rate; DM, distant metastasis rate; GCT, germ cell tumor; YST, yolk sac tumor; EC, embryonal carcinoma; CHC, choriocarcinoma.

**Table 2:** Organotropic metastasis rates of malignant germ cell tumors according to histologic subtype and primary site.

	Bone	Brain	Liver	Lung	Other
Testicular GCTs					
Seminoma	15.3% (60/392)	3.3% (13/393)	16.6% (65/392)	46.7% (184/394)	50.4% (143/284)
YST	17.3% (18/104)	10.7% (11/103)	34.0% (36/106)	73.6% (78/106)	34.3% (23/67)
Teratoma	6.5% (4/62)	9.7% (6/62)	31.7% (19/60)	80.3% (49/61)	18.8% (6/32)
EC	7.0% (24/344)	4.4% (15/342)	12.5% (43/344)	91.4% (317/347)	15.1% (31/205)
CHC	6.9% (14/204)	32.5% (67/206)	37.6% (77/205)	94.2% (194/206)	24.1% (26/108)
Mixed GCT	7.8% (92/1182)	8.2% (97/1180)	19.0% (224/1181)	91.9% (1093/1189)	16.9% (120/711)
Ovarian GCTs					
Dysgerminoma	9.1% (1/11)	0% (0/11)	8.3% (1/12)	36.4% (4/11)	58.3% (7/12)
YST	6.5% (3/46)	0% (0/46)	57.4% (27/47)	15.2% (7/46)	62.9% (22/35)
Teratoma	14.3% (2/14)	0% (0/14)	42.9% (6/14)	28.6% (4/14)	84.6% (11/13)
EC	0% (0/1)	0% (0/1)	100% (1/1)	0% (0/1)	100% (1/1)
CHC	0% (0/7)	14.3% (1/7)	62.5% (5/8)	28.6% (2/7)	83.3% (5/6)
Mixed GCT	9.11% (2/22)	0% (0/22)	45.5% (10/22)	50.0% (11/22)	50.0% (9/18)
Mediastinal GCTs					
Seminoma	18.8% (3/16)	6.3% (1/16)	18.8% (3/16)	31.3% (5/16)	71.4% (5/7)
YST	42.3% (11/26)	11.5% (3/26)	51.9% (14/27)	72.0% (18/25)	33.3% (5/15)
Teratoma	25.0% (1/4)	25.0% (1/4)	25.0% (1/4)	75.0% (3/4)	0.0% (0/1)
EC	0.0% (0/3)	0.0% (0/3)	0.0% (0/3)	100.0% (3/3)	0.0% (0/3)
CHC	12.0% (3/25)	56.0% (14/25)	52.0% (13/25)	96.0% (24/25)	53.3% (8/15)
Mixed GCT	27.6% (8/29)	3.4% (1/29)	24.1% (7/29)	55.2% (16/29)	47.6% (10/21)

GCT, germ cell tumor; YST, yolk sac tumor; EC, embryonal carcinoma; CHC, choriocarcinoma.

## Data Availability

The data presented in this study are available on request from the corresponding author.

## Supplementary Materials

The following supporting information can be downloaded at: https://www.mdpi.com/article/10.3390/medicina61111990/s1, Table S1. Sensitivity analysis restricted to cases diagnosed between 2016 and 2022, when complete data on all metastatic sites were available. The trends observed were consistent with those from the full 2010–2022 cohort, confirming the robustness of the findings.; Table S2. Sensitivity analysis comparing 5-year and 8-year age cutoffs for the classification of prepubertal and postpubertal germ cell tumors.; Table S3. SEER*Stat Workflow for Case Selection and Data Extraction; STROBE Checklist.

## Abbreviations

The following abbreviations are used in this manuscript:

GCT	Germ cell tumor
YST	Yolk sac tumor
EC	Embryonal carcinoma
CHC	Choriocarcinoma
GCNIS	Germ cell neoplasia in situ
WHO	World Health Organization
SEER	Surveillance, Epidemiology, and End Results
