# Supplementary material for: Metastatic Patterns of Malignant Germ Cell Tumors Vary by Histologic Subtype and Primary Site
Source: Medicina (Kaunas). 2025 Nov 5;61(11):1990. doi: 10.3390/medicina61111990 (PMC12654695; doi:10.3390/medicina61111990)
Supplement: Supplementary file 1 [file medicina-61-01990-s001.zip › Table S3.pdf]

Supplementary Table S3. SEER*Stat Workflow for Case Selection and Data Extraction

Step	Action	Details
1	Launch SEER*Stat (Version 9.*) and create a new Case Listing Session	• Open SEER*Stat (version 9.x) → From menu, select New Session > Case Listing.<br>• If the Client–Server Login window appears, enter the username and password you received when registering for SEER and click Log In.
2	Database Selection	• Choose “Incidence – SEER Research Data, 17 Registries, Nov 2024 Sub (2000–2022)”
3	Case Selection – Primary Sites	• Click the “Edit…” button under the “Selection Statement” section of the Selection tab.<br>• In the newly opened Case Selection window, click “+ New Line.”<br>• A new Case Selection Line window will appear. From the list of variable categories, select “Site and Morphology” and then choose “Site recode ICD-O-3/WHO 2008.” A list of corresponding values will appear in the Values panel on the right. From this list, select “Trachea, Mediastinum and Other Respiratory Organs,” “Ovary,” and “Testis” to include mediastinal, ovarian, and testicular primary sites.<br>• Finally, click the “OK” button at the lower right corner of the window.
4	Case Selection – Histology	• In the Case Selection window, click “+ New Line” again.<br>• A new Case Selection Line window will appear. As in Step 3, select “Site and Morphology” from the list of variable categories, then choose “ICD-O-3 Hist/behav, malignant.”.<br>• A list of available values will appear in the Values panel on the right. From this list, select “9060/3: Dysgerminoma,” “9061/3: Seminoma, NOS,” “9070/3: Embryonal carcinoma, NOS,” “9071/3: Yolk sac tumor,” “9080/3: Teratoma, malignant, NOS,” “9085/3: Mixed germ cell tumor,” and “9100/3: Choriocarcinoma, NOS.”<br>• Click the “OK” button at the lower right corner of the window.<br>• Click the “OK” button at the lower right corner of the Case Selection window once more to complete the case selection.

5	Variables to Include in Output	· Go to the Table tab and, under the Available Variables section, select the variables to be extracted.<br>· The variables to include are as follows:<br>▷ Patient ID (Other > Patient ID)<br>▷ Sex (Race, Sex, Year Dx > Sex)<br>▷ Year of diagnosis (Race, Sex, Year Dx > Year of diagnosis)<br>▷ Age at diagnosis (Race and Age (case data only) > Age recode with single ages and 85+)<br>▷ Primary site (Site and Morphology > Site recode ICD-O-3/WHO 2008)<br>▷ Histologic subtype (Site and Morphology > ICD-O-3 Hist/behav, malignant)<br>▷ Presence of bone metastasis (Extent of Disease > SEER Combined Mets at DX-bone (2010+))<br>▷ Presence of brain metastasis (Extent of Disease > SEER Combined Mets at DX-brain (2010+))<br>▷ Presence of liver metastasis (Extent of Disease > SEER Combined Mets at DX-liver (2010+))<br>▷ Presence of lung metastasis (Extent of Disease > SEER Combined Mets at DX-lung (2010+))<br>▷ Presence of Other metastasis (Extent of Disease > Mets at DX-Other (2016+))<br>▷ TNM stage (Stage – 6th edition > Derived AJCC T, 6th ed (2004-2015), Derived AJCC N, 6th ed (2004-2015), Derived AJCC M, 6th ed (2004-2015) and Stage – 7th edition > Derived AJCC T, 7th ed (2010-2015), Derived AJCC N, 7th ed (2010-2015), Derived AJCC M, 7th ed (2010-2015), Derived SEER Combined T (2016-2017), Derived SEER Combined N (2016-2017), Derived SEER Combined M (2016-2017) and Stage – 8th edition > Derived EOD 2018 T Recode (2018+), Derived EOD 2018 N Recode (2018+), Derived EOD 2018 M Recode (2018+))<br>· After selecting each variable, click the “Column” button. When the variable appears in the Columns list at the top, it has been successfully selected.
6	Export Data	· From the menu, select Actions > Execute to run the data extraction.<br>· Once the extraction is complete, choose Export to save the data as a file for further processing. Convert to Excel or CSV if desired.
7	Statistical Analysis	· Import data.<br>· Perform Pearson’s chi-square or Fisher’s exact test for categorical comparisons.
