# Supplementary material for: Metastatic Patterns of Malignant Germ Cell Tumors Vary by Histologic Subtype and Primary Site
Source: Medicina (Kaunas). 2025 Nov 5;61(11):1990. doi: 10.3390/medicina61111990 (PMC12654695; doi:10.3390/medicina61111990)
Supplement: Supplementary file 1 [file medicina-61-01990-s001.zip › STROBE Checklist.pdf]

STROBE Checklist: Cohort Studies

Title: Metastatic Patterns of Malignant Germ Cell Tumors Vary by Histologic Subtype and Primary Site

Study Design: Population-based retrospective cohort study using SEER data (2010–2022)

Author: [Your Name]

Item No.	Section/Topic	Recommendation	Reported on page/section
1	Title and abstract	Indicate the study design with a commonly used term and summarize what was done and what was found.	Title, Abstract
2	Background/rationale	Explain the scientific background and rationale for the investigation being reported.	Introduction
3	Objectives	State specific objectives, including any prespecified hypotheses.	Introduction
4	Study design	Present key elements of study design early in the paper.	Methods (2.1 Data Source and Patient Selection)
5	Setting	Describe the setting, locations, and relevant dates, including periods of recruitment, exposure, follow-up, and data collection.	Methods (2.1)
6	Participants	Give the eligibility criteria, sources, and methods of selection of participants.	Methods (2.1)
7	Variables	Clearly define all outcomes, exposures, predictors, potential confounders, and effect modifiers.	Methods (2.2 Data Processing and Analysis)
8	Data sources/measurement	For each variable, give sources of data and details of methods of assessment (measurement).	Methods (2.1, 2.2)

9	Bias	Describe any efforts to address potential sources of bias.	Discussion (Limitations)
10	Study size	Explain how the study size was arrived at.	Methods (2.1)
11	Quantitative variables	Explain how quantitative variables were handled in the analyses (e.g., groupings, cutoffs).	Methods (2.2)
12	Statistical methods	Describe all statistical methods, including those used to control for confounding, subgroup analyses, and sensitivity analyses.	Methods (2.2 Statistical Analysis); Supplementary Tables S1–S2
13	Participants	Report numbers of individuals at each stage of study (e.g., eligible, included, analyzed).	Results (3.1)
14	Descriptive data	Give characteristics of study participants (e.g., demographics, clinical features).	Results (Table 1)
15	Outcome data	Report numbers of outcome events or summary measures over time.	Results (Tables 2–3, Figures 1–3)
16	Main results	Give unadjusted estimates and, if applicable, adjusted estimates and their precision (e.g., 95% CI). Make clear which confounders were adjusted for and why.	Results, Discussion
17	Other analyses	Report subgroup analyses, sensitivity analyses, and interactions, if done.	Supplementary Tables S1–S2
18	Key results	Summarize key results with reference to study objectives.	Discussion
19	Limitations	Discuss limitations of the study, taking into account potential bias or imprecision.	Discussion (Limitations)
20	Interpretation	Give a cautious overall interpretation of results considering objectives,	Discussion

		limitations, and other relevant evidence.	
21	Generalizability	Discuss the generalizability (external validity) of the study results.	Discussion
22	Funding	Give the source of funding and the role of the funders for the present study.	Funding: None
